# Baicalin suppresses colorectal cancer proliferation and induces M1 polarization of tumor-associated macrophages by promoting proteasomal degradation of HK2

**DOI:** 10.3389/fimmu.2026.1812964

**Published:** 2026-05-04

**Authors:** Dong-hui Bai, Duan Gao, Ying Xiong, Yu-Ling Chang, Xia Gan, Li Yang, Pan-pan Wang, Rong-hua Zhang

**Affiliations:** 1 College of Traditional Chinese Medicine, Jinan University, Guangzhou, China; 2 College of Pharmacy, Jinan University, Guangzhou, China; 3 Department of Chinese Medicine, The First Affiliated Hospital of Jinan University, Guangzhou, China

**Keywords:** baicalin, cGAS/STING, colorectal cancer, hexokinase II, tumor-associated macrophage

## Abstract

Colorectal cancer (CRC) ranks as the third most diagnosed cancer and the second leading cause of cancer-related mortality globally. Hexokinase II, a key rate-limiting enzyme in tumor glycolysis, is an important therapeutic target. In this study, we report that baicalin, a flavonoid derived from *Scutellaria baicalensis*, acts as a HK2 inhibitor and exerts anti–colorectal cancer activity. *In vitro*, baicalin markedly suppressed colorectal cancer cell proliferation and colony formation. Molecular docking, molecular dynamics simulations, DARTS, and CETSA suggest an association between baicalin and HK2, while MG132 rescue and HK2 immunoprecipitation indicate that baicalin promotes ubiquitination-associated proteasomal degradation of HK2. Mechanistically, baicalin inhibits HK2, reduces glycolysis, and causes mitochondrial damage, thereby activating the cGAS/STING innate immune signaling pathway and increasing IFN-β production. IFN-β contributes to reshaping the tumor immune microenvironment. In an MC38 syngeneic tumor model, baicalin significantly inhibited tumor growth, reduced HK2 protein levels, activated the cGAS/STING pathway, and promoted a shift in tumor-associated macrophages toward an M1-like polarization state. Collectively, this study uncovers a novel strategy for targeting HK2 to regulate both tumor cell metabolism and the immune microenvironment, providing a potential therapeutic approach for colorectal cancer.

## 1 Introduction

Colorectal cancer (CRC) ranks as the third most diagnosed cancer and the second leading cause of cancer-related mortality globally (1–3). The worldwide burden of CRC is projected to rise markedly by 2040, reaching about 3.2 million new diagnoses and 1.6 million deaths (4). At present, therapeutic strategies for CRC include surgery, chemotherapy, radiotherapy, and targeted therapy. However, clinical outcomes remain limited by systemic toxicity, drug resistance, and inadequate tumor targeting, resulting in suboptimal overall efficacy (5, 6). Therefore, developing novel therapeutic strategies to overcome these limitations has become a major priority in CRC research.

Dysregulated energy metabolism is a fundamental characteristic of cancer, playing a crucial role in tumor progression (6, 7). Tumor cells alter their energy metabolism pathways, particularly by enhancing glycolysis, to support rapid proliferation, sustained growth, and metastasis (8–10). Excessive glycolytic activity not only supplies tumor cells with essential energy but also produces a range of metabolic byproducts that enable their survival in hostile microenvironments (11–13). Hexokinase 2 (HK2) catalyzes the formation of glucose-6-phosphate from glucose, thereby controlling the initial step of glycolysis and promoting tumor cell survival, growth, and metabolism (14–16). Studies indicate that HK2 is frequently upregulated in multiple cancer types, and higher HK2 expression is strongly correlated with tumor growth, metastasis, and poor prognosis (17–19). Beyond glycolysis, HK2 associates with VDAC1 at the mitochondrial surface to stabilize mitochondria and regulate mPTP opening (20–22). Therefore, given its multifaceted roles in tumor cell metabolism and survival, HK2 represents a promising therapeutic target in cancer. Baicalin, a flavonoid derived from *Scutellaria baicalensis* roots, has been widely studied for its notable antioxidant, anti-inflammatory, and anticancer properties (23–25). Multiple studies have demonstrated that baicalin can efficiently suppress the growth of various cancer cell lines and induce cell apoptosis (26, 27). Studies have shown that baicalin can attenuate immune evasion in oral squamous cell carcinoma by decreasing lactate levels in the tumor microenvironment (25). In addition, baicalin exhibits anti-osteosarcoma effects by modulating the Nrf2/xCT/GPX4 ferroptosis signaling axis (28). Previous research indicates that baicalin inhibits IFN-I–induced glycolysis in neutrophils by downregulating the PKC/Raf/MEK/ERK and PI3K/AKT pathways, as well as reducing the expression of HK2, HK3, PKM2, and LDHA (29). The effects of baicalin on CRC and its influence on glycolysis remain inadequately explored. In this study, we show that baicalin engages HK2 and promotes its proteasome-dependent degradation, consistent with ubiquitination-associated regulation, which is accompanied by reduced glycolysis, mitochondrial damage, and suppression of CRC cell proliferation. Moreover, baicalin-induced mitochondrial injury activates the cGAS/STING signaling pathway, which subsequently drives M1-like polarization of tumor-associated macrophages.

## 2 Materials and methods

### 2.1 Reagents

Baicalin and oxaliplatin, both with a purity of ≥98%, were sourced from Beijing Solarbio Science & Technology Co., Ltd. (Beijing, China). DMEM, PBS, FBS, and trypsin were all sourced from Gibco (Thermo Fisher Scientific, Waltham, MA, USA). CCK-8, BCA, 2-NBDG, ATP, L-Lactate, and CO-IP assay kit were purchased from Beyotime Biotechnology (Shanghai, China). The 2’3’-cGAMP and IFN-β ELISA kits were sourced from Jiangsu Ebiom Industrial Co., Ltd. (Jiangsu, China). Primary antibodies against HK2 (#2867), p-TBK1 (#5483), TBK1 (#3504), p-STING (#72971), STING (#13647), p-IRF3 (#29047), IRF3 (#4302), CD206 (#24595), Arg1 (#93668), CD86 (#19589), iNOS (#13120), β-Actin (#4970), and GAPDH (#2118) were obtained from Cell Signaling Technology (CST; Beverly, MA, USA).

### 2.2 Cell culture

HCT116 and MC38 cells were obtained from the Cell Bank of the Shanghai Institutes for Biological Sciences, Chinese Academy of Sciences (Shanghai, China). Cells were maintained in DMEM supplemented with 10% fetal bovine serum and 1% penicillin–streptomycin, and incubated at 37 °C in a humidified atmosphere containing 5% CO_2_.

### 2.3 CCK-8 assay

The Cell Counting Kit-8 (CCK-8) assay was employed to evaluate cell viability. HCT116 and MC38 cells were plated into 96-well plates at 3,000 cells per well. After attachment, cells were treated with various concentrations of baicalin (0, 10, 20, or 40 µmol/L) for 24, 48, or 72 h. Next, 10 µL CCK-8 reagent was introduced into every well, and maintained at 37 °C for 1 h. Absorbance was determined at 450 nm with a Bio-Rad microplate reader.

### 2.4 Cell transfection

siRNA transfection was carried out following the manufacturer’s protocol using Lipofectamine 2000 (11668019, Invitrogen, USA), followed by analysis 48 h post-transfection. The sequences used for transfection were provided in **Table 1**.

**Table 1:** siRNA sequences.

Target	Strand	Sequence (5’→3’)
Human siHK2-1	Sense	GCCUGGCUAACUUCAUGGAUATT
	Antisense	UAUCCAUGAAGUUAGCCAGGCTT
Human siHK2-2	Sense	ACUGAGUUUGACCAGGAGAUUTT
	Antisense	AAUCUCCUGGUCAAACUCAGUTT
Mouse siHK2-1	Sense	GCCGUGGUAAAUGACACAGUUTT
	Antisense	AACUGUGUCAUUUACCACGGCTT
Mouse siHK2-2	Sense	CGGUACAGAGAAAGGAGACUUTT
	Antisense	AAGUCUCCUUUCUCUGUACCGTT

To generate HK2-overexpressing cells, the full-length HK2 cDNA was cloned into a lentiviral expression vector. To generate HK2-knockdown cells, shRNA targeting HK2 was designed based on the validated siHK2–2 sequence and cloned into a lentiviral vector. HEK293T cells were co-transfected with the recombinant plasmid using Lipofectamine 3000 (L3000015, Invitrogen, USA). The culture supernatants were collected after 48 h, and the virus was concentrated by polyethylene glycol (PEG) precipitation to yield high-titer lentiviral particles. Target cells were infected with the concentrated virus, and stable HK2-overexpressing or HK2-knockdown cell lines were generated by selecting with puromycin (2.5 μg/mL) for 7 days in complete medium.

### 2.5 Colony formation assay

HCT116 and MC38 cells were cultured in 6-well plates (1,000 cells per well) and exposed to baicalin. After 14 days, the cultures were gently rinsed with PBS, then fixed with 500 µL of 4% paraformaldehyde for 20 min. Finally, colonies were stained with 0.5% crystal violet.

### 2.6 2-NBDG fluorescent glucose uptake assay

HCT116 and MC38 cells were plated in 6-well plates at 5 × 10^5^ cells per well. Following baicalin treatment, cells were rinsed with PBS and incubated in glucose-free complete medium for 6 h. Afterward, the cell pellet was collected. Following the glucose uptake fluorescence assay kit protocol (S0561M, Beyotime, China), 1 mL of 1×2-NBDG working solution was applied, and cells were cultured at 37 °C for 60 min. After incubation, cells were centrifuged to discard the supernatant and washed once with PBS. Finally, cell fluorescence intensity was analyzed using flow cytometry (BD Biosciences, USA) with excitation at 488 nm and emission at 542 nm.

### 2.7 ATP content detection

All steps were performed following the ATP assay kit protocol (S0026, Beyotime, China). A total of 200 μL of lysis buffer was added to each well of a 6-well plate and incubated on ice for 10 min. The lysates were centrifuged at 12,000 × g for 5 min at 4 °C, and the supernatant was collected. Subsequently, 100 μL of the detection reagent was added to each well and incubated for 3–5 min at room temperature to eliminate background ATP. Then, 20 μL of the sample or ATP standard was added, gently mixed, and the luminescence (RLU) was recorded. ATP content was calculated based on the standard curve, and protein concentration was determined using a BCA assay. Finally, ATP concentration was normalized and expressed as nmol/μg protein.

### 2.8 L-Lactate content assay

Following the protocol of the L-Lactate content detection kit (S0208S, Beyotime, China), 100 μL of lysis buffer was added per 1 × 10^6^ cells, and the cells were incubated on ice for 10 min to complete lysis. The lysates were centrifuged at 12,000 × g for 5 min at 4 °C, and the supernatant was collected for analysis. 50 μL of the supernatant was added to a 96-well plate, and blank control wells were set up. Subsequently, 50 μL of the WST-8 working solution was added to each well, gently mixed, and incubated at 37 °C for 30 min. Absorbance at 450 nm was measured. The zero-concentration standard signal was subtracted from all standard wells, and the blank signal was subtracted from all sample wells. Based on the corrected signals, a standard curve was generated, and the L-lactate concentration in the samples was calculated.

### 2.9 Mitochondrial membrane potential detection

HCT116 and MC38 cells were inoculated in 6-well plates at a density of 5 × 10^5^ cells per well. After treatment, the mitochondrial membrane potential was measured following the manufacturer’s protocol (C2006, Beyotime, China). The JC-1 staining working solution was prepared by adding 5 μL of JC-1 (200x) to 1 mL of staining buffer. Cell pellets were collected and resuspended in 1 mL of the JC-1 staining working solution, and mixed thoroughly. Cells were preserved at 37 °C for 20 min, rinsed with 1× JC-1 staining buffer, and then harvested and resuspended in 500 μL of the same buffer. Flow cytometry was utilized to measure fluorescence, with red fluorescence excited at 488 nm and emitting at 590 nm, and green fluorescence excited at 488 nm and emitting at 530 nm. The mitochondrial membrane potential was determined by calculating the ratio of red to green JC-1 fluorescence intensity.

### 2.10 Mitochondrial permeability transition pore assay

The culture medium was removed, and the cells were washed once with PBS. In accordance with the instructions of the mPTP assay kit (C2009S, Beyotime, China), 500 µL of Calcein AM staining solution and 5 µL of CoCl_2_ solution were added and mixed thoroughly. The cells were incubated at 37 °C in the dark for 30 min. Afterward, the medium was replaced with pre-warmed fresh culture medium, and the cells were further incubated at 37 °C for an additional 30 min to allow complete hydrolysis of Calcein AM into green fluorescent calcein. Subsequently, the cells were washed twice with PBS, and the nuclei were stained with DAPI. Finally, the cells were observed under a laser scanning confocal microscope (Olympus, Japan) with excitation at 494 nm and emission at 517 nm.

### 2.11 HK2 expression and prognosis in CRC

Firstly, the GEPIA database was used to assess HK2 expression differences in colorectal adenocarcinoma (COAD) and rectal adenocarcinoma (READ) relative to normal tissues. Using the Kaplan–Meier Plotter database, CRC patients were divided into high and low HK2 expression groups based on the median expression level of HK2. Kaplan-Meier curves for overall survival (OS) were plotted, and the hazard ratio (HR) and Log-rank P value were computed to evaluate the relationship between HK2 expression and patient prognosis.

### 2.12 IFN-β measurement

After collecting the supernatants from HCT116 and MC38 cells, IFN-β levels were measured using an IFN-β ELISA kit. Samples and standards were loaded into the pre-coated ELISA plate wells, then the kit-provided enzyme-conjugated secondary antibody and substrate were added sequentially. Absorbance was determined at 450 nm using a Bio-Rad microplate reader.

### 2.13 2’3’-cGAMP measurement

The cell lysates and standards were added to the pre-coated antibody ELISA plate. Subsequently, the enzyme-conjugated secondary antibody and substrate were added according to the manufacturer’s instructions. The plate was incubated under the recommended conditions. After incubation, absorbance at 450 nm was measured, and the 2′3′-cGAMP concentration was calculated based on the standard curve.

### 2.14 Cellular thermal shift assay

Based on previous studies (30, 31), equal numbers of cells were treated with or without 40 μmol/L baicalin for 3 h. After collection, the cells were resuspended in PBS containing PMSF and aliquoted into six tubes. The samples were subjected to a temperature gradient (37, 42, 47, 52, 57, and 62 °C) using a PCR instrument, with thermal treatment applied for 3 min at each target temperature followed by rapid cooling. Subsequently, cells were exposed to three freeze–thaw cycles (using liquid nitrogen followed by a 37 °C water bath) to achieve complete lysis. The lysates were then centrifuged at 12,000 rpm for 10 min at 4 °C, and the supernatants containing soluble proteins were harvested. Equal amounts of protein were used for subsequent western blot. For thermal melting curve analysis, protein abundance at each temperature point was quantified by western blot densitometry. Relative soluble protein levels were normalized to the lowest temperature condition (37 °C) and plotted against temperature. The resulting melting curves were fitted using a sigmoidal Boltzmann equation to determine the apparent melting temperature shift (ΔTm).

For isothermal CETSA, cells were treated with increasing concentrations of baicalin for 3 h and then exposed to a fixed temperature of 52 °C for 3 min. After thermal treatment, samples were cooled, lysed, and centrifuged as described above. The resulting supernatants containing soluble proteins were collected for western blot analysis. For dose–response analysis, band intensities from isothermal CETSA were quantified, normalized to control, and fitted using a sigmoidal dose–response (variable slope, 4PL) model to calculate EC50 values. All curve fitting was performed using nonlinear regression in GraphPad Prism.

### 2.15 Drug affinity responsive target stability assay

The experiment was conducted following the DARTS kit instructions (S3206S, Beyotime, China). Cells were lysed with 100 μL of lysis buffer per 1 × 10^6^ cells and incubated at 4 °C for 20 min. The lysates were then centrifuged at 12,000 rpm for 10 min, and the supernatants were collected. Protein concentrations were determined using a BCA assay. Based on the quantification results, protein samples were adjusted to a final concentration of approximately 2 mg/mL. The resulting protein extracts were mixed with an equal volume of DMSO or with various concentrations of baicalin and incubated at room temperature for 1 h. A nonspecific protease (streptozyme) was then added at a 1:1000 ratio (protease:total protein), and the samples were incubated for an additional 30 min. The reaction was terminated by the immediate addition of Protease Inhibitor Cocktail (100×). Subsequently, protein concentrations were determined using a BCA assay. Samples were mixed with 5× SDS-PAGE loading buffer, heated at 100 °C for 10 min, and subjected to western blot analysis with 20 μg of total protein per well.

### 2.16 Molecular docking

The receptor structure was prepared in PyMOL 2.6.0 by removing solvent molecules. Hydrogen atoms were added, and partial charges were assigned in AutoDock Tools. The receptor and ligand were subsequently exported in .pdbqt format. Docking was carried out using AutoDock Vina, and the resulting binding poses were visualized in PyMOL.

### 2.17 Molecular dynamics simulation

Molecular dynamics (MD) simulations were performed using GROMACS 2023.4 for 100 ns on protein–ligand complexes derived from docking. Protein topology was generated with pdb2gmx using the Amber14SB force field, and ligand parameters were assigned with GAFF2 via AmberTools22. The system was solvated in a TIP3P water box with a 1.2 nm buffer, neutralized with Na^+^/Cl^−^ ions, and treated with PME for long-range electrostatics. After energy minimization using the steepest descent algorithm, the system was heated to 300 K under NVT conditions and equilibrated at 300 K and 1 bar under NPT conditions. A 100 ns production run was then conducted without restraints. Trajectory analysis included RMSD, RMSF, and radius of gyration (Rg). Free energy landscapes (FEL) were generated using g_sham and xpm2txt to evaluate conformational stability.

### 2.18 Co-immunoprecipitation assay

Cells were treated with baicalin in the presence or absence of MG132 for 24 h and then were lysed on ice for 30 min using RIPA buffer. After centrifugation, the supernatants were collected as total protein samples. A fraction of the lysate was set aside as the Input control, and the remaining sample was used for Co-IP assay. Specifically, the HK2-specific antibody was preincubated with Protein A/G magnetic beads at room temperature for 1 h to allow antibody–bead coupling. In parallel, normal IgG was used as a negative control to assess non-specific binding. The total protein lysate was then mixed with the antibody–bead complex and incubated overnight at 4 °C with gentle agitation. The next day, the antibody–bead complexes were collected using a magnetic separator, followed by several washes with lysis buffer to reduce non-specific binding. The bead–protein complexes were then resuspended in SDS loading buffer and heated at 100 °C for 10 min to elute bound proteins. The resulting samples were analyzed by western blot. For detection, the ubiquitination level of HK2 was assessed by western blot using an anti-ubiquitin antibody.

### 2.19 Western blot

HCT116 and MC38 cells (5 × 10^5^ per well) were plated in 6-well plates and treated with baicalin for 24 h. After treatment, cells were lysed on ice for 20 min using 200 μL of RIPA lysis buffer (P0013B, Beyotime, China) with 1% PMSF (ST506, Beyotime, China) and 1% phosphatase inhibitor (P1082, Beyotime, China). The lysates were centrifuged at 12,000 rpm for 10 min at 4 °C, and the supernatant was harvested. Protein levels were quantified using a BCA assay (P0010, Beyotime, China). Equal protein quantities (20 μg) were separated by 10% SDS-PAGE, and transferred to a PVDF membrane via wet transfer at 300 mA, 4 °C for 60 min. The membrane was blocked using 5% non-fat milk at room temperature for 1 h, followed by an overnight incubation at 4 °C with primary antibodies at a 1:1000 dilution. The following day, the membrane underwent three TBST washes, and was incubated with HRP-conjugated secondary antibody (1:2000 dilution) at room temperature for 1 h. After additional washes with TBST, the membrane was exposed to an ECL chemiluminescent detection reagent (MA0186-2, Meilunbio, China), and images were obtained with a Bio-Rad imaging system (exposure time: 30 s to 5 min). Finally, band intensity was analyzed using Image Lab software.

### 2.20 RT-qPCR

Total RNA was isolated using Trizol reagent (15596018, Thermo Fisher Scientific, USA) and subsequently reverse transcribed into cDNA using a reverse transcription kit (AG11711, Accurate Biology, China). Gene-specific primers were prepared, and qPCR was carried out using SYBR Green chemistry. Relative mRNA expression was quantified using the following cycling protocol: 95 °C for 30 s, followed by 40 cycles of 95 °C for 5 s and 60 °C for 30 s. Each experiment was conducted in triplicate. Relative mRNA expression was quantified using the 2^(-ΔΔCt) method, with GAPDH and β-actin serving as internal references for normalization. Refer to **Table 2** for the primer sequences.

**Table 2:** Primer sequences.

Gene	Primer type	Sequence (5’→3’)
Human-HK2	Forward	GATTGCCTCGCATCTGCTTG
	Reverse	GCTCCAAGCCCTTTCTCCAT
Mouse-HK2	Forward	GCGTGGATGGCTCTGTCTACAAG
	Reverse	GGAGGAAGCGGACATCACAATCG
Mouse-CD206	Forward	CTCGGGACTCTGGATTGGACTC
	Reverse	TGATGATGGACTTCCTGGTAGCC
Mouse-Arg1	Forward	AAGACAGCAGAGGAGGTGAAGAG
	Reverse	GGTAGTCAGTCCCTGGCTTATGG
Mouse-CD163	Forward	AATCACATCATGGCACAGGTCAC
	Reverse	TCGTCGCTTCAGAGTCCACAG
Mouse-IL-10	Forward	GTTGCCAAGCCTTATCGGAAATG
	Reverse	TCTCACCCAGGGAATTCAAATGC
Mouse-CD86	Forward	TCTGCCGTGCCCATTTACAAAG
	Reverse	GTGCCCAAATAGTGCTCGTACAG
Mouse-iNOS	Forward	TCACTCAGCCAAGCCCTCAC
	Reverse	TCCAATCTCTGCCTATCCGTCTC
Mouse-TNF-α	Forward	CACGCTCTTCTGTCTACTGAACTTC
	Reverse	CTTGGTGGTTTGTGAGTGTGAGG
Mouse-GAPDH	Forward	GGCAAATTCAACGGCACAGTCAAG
	Reverse	TCGCTCCTGGAAGATGGTGATGG
Mouse-β-actin	Forward	TACTGCTCTGGCTCCTAGCA
	Reverse	CGGACTCATCGTACTCCTGC
Human-GAPDH	Forward	TGACATCAAGAAGGTGGTGAAGCAG
	Reverse	GTGTCGCTGTTGAAGTCAGAGGAG
Human-β-actin	Forward	ATTGCCGACAGGATGCAGAA
	Reverse	CGGACTCGTCATACTCCTGC

### 2.21 Macrophage polarization assay

Bone marrow cells were harvested from mouse femurs and tibias. After red blood cell lysis, bone marrow–derived mononuclear cells were collected, seeded in 6-well plates at a density of 2×10^6^ cells per well, and cultured in complete MEM supplemented with 20 ng/mL M-CSF for 7 days to differentiate into bone marrow–derived macrophages (BMDMs). For Transwell co-culture, BMDMs were plated in the lower chamber, while MC38 cells subjected to three conditions (shNC MC38, shHK2 MC38, and MC38 pretreated with 40 μmol/L baicalin for 48 h) were placed in the upper chamber of a Transwell system with a 0.4 μm pore-size membrane. Co-culture was maintained for 48 h. BMDMs from the lower chamber were then harvested, and total protein and RNA were isolated for subsequent western blot and RT-qPCR analyses.

### 2.22 Animals and treatments

The animal studies adhered to guidelines approved by Jinan University’s Animal Ethics Committee. Six-week-old male C57BL/6 mice were obtained from Guangdong Yaokang Biotechnology Co., Ltd. and housed under SPF conditions at the Animal Experimental Center of Jinan University. To establish a CRC syngeneic model, 5×10^5^ MC38 cells were subcutaneously injected into the right flank of each mouse. Mice were then randomly distributed into four groups (n=5): control, baicalin low-dose group (20 mg/kg, Baicalin-L), baicalin high-dose group (40 mg/kg, Baicalin-H), and oxaliplatin (0.5 mg/kg, positive control). Treatments were administered daily via oral gavage for 21 consecutive days. Tumor size was recorded every 3 days, and tumor volume was calculated using the formula V = length × width^2^ × 0.5. At the conclusion of the experiment, mice were euthanized, and tumors were harvested and weighed.

### 2.23 Flow cytometric analysis of tumor tissue

Fresh mouse tumor tissue was minced and digested with collagenase IV at 37 °C for 30 min. After treatment with red blood cell lysis buffer, the mixture was filtered through a 70 μm cell strainer to create a single-cell suspension. Cells were centrifuged, resuspended in 100 μL PBS, and incubated with 5 μL anti-mouse CD16/CD32 (E-AB-F0997A, Elabscience, China) to block Fc receptors for 15 min. Subsequently, 5 μL anti-mouse F4/80-PE (F2148002, MultiSciences, China), 5 μL anti-mouse CD11b-PerCP (E-AB-F1081F, Elabscience, China), 5 μL anti-mouse CD86-FITC (F2108601, MultiSciences, China), and 5 μL anti-mouse CD206-APC (F2120603, MultiSciences, China) were added for macrophage labeling and incubated at room temperature in the dark for 30 min. Following staining, cells were washed with PBS to eliminate unbound antibodies and resuspended in 500 μL PBS. DAPI was added to differentiate live from dead cells. Cell analysis was conducted using a BD FACSCanto flow cytometer (BD Biosciences, USA), and the results were processed using FlowJo software. FSC/SSC gating was initially applied to exclude cell debris, followed by dead cell exclusion using DAPI staining. F4/80+CD11b+ cells were categorized as macrophages, and macrophage polarization was evaluated based on the CD86/CD206 dual staining. Macrophage phenotypic distribution was assessed by CD86/CD206 dual staining. Using an operational marker-based classification, we defined and analyzed CD206+CD86− and CD206−CD86+ populations as distinct macrophage phenotypes.

### 2.24 Hematoxylin and eosin staining

Tissues were fixed in 4% paraformaldehyde, then dehydrated and embedded in paraffin. Sections underwent deparaffinization, rehydration, hematoxylin and eosin staining, dehydration, clearing, and were subsequently examined under a light microscope.

### 2.25 Immunohistochemical staining

Paraffin-embedded sections were first deparaffinized and rehydrated, followed by antigen retrieval using citrate buffer. Endogenous peroxidase activity was inhibited with 3% H2O2, and nonspecific binding was blocked using BSA. The sections were then incubated with a primary antibody, followed by an HRP-conjugated secondary antibody. Staining was visualized with DAB, followed by hematoxylin counterstaining. Slides were dehydrated, cleared, mounted, and examined under a microscope.

### 2.26 Statistical analysis

All data were expressed as mean ± SEM, and analyzed utilizing GraphPad Prism 9.0 software. Two-group comparisons utilized a two-tailed Student’s t-test, whereas multiple-group comparisons were conducted using one-way ANOVA followed by an appropriate *post hoc* test. Significance levels are denoted as follows: * *p* < 0.05, ** *p* < 0.01, *** *p* < 0.001, and **** *p* < 0.0001.

## 3 Results

### 3.1 Baicalin inhibits CRC cell proliferation by targeting HK2

**Figure 1:**
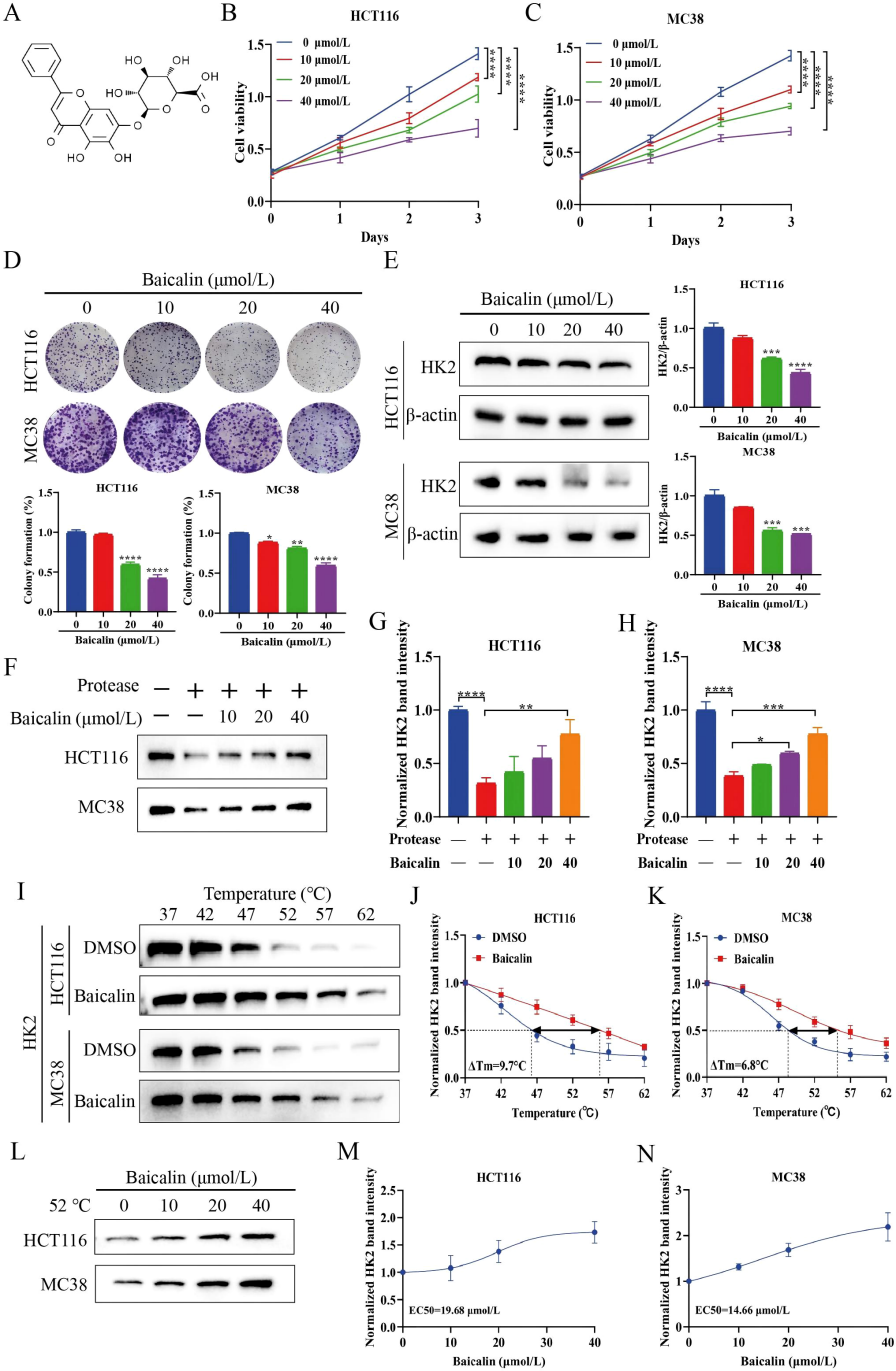
Baicalin engages HK2 to inhibit CRC cell proliferation. **(A)** Chemical structure of baicalin. **(B, C)** Cell viability (n=6). **(D)** Colony formation assay (n=3). **(E)** Western blot analysis of HK2 expression (n=3). **(F)** DARTS assay (n=3). **(G, H)** Quantification of HK2 levels in the DARTS assay. **(I)** CETSA analysis (n=3). **(J, K)** Quantification of HK2 levels in the CETSA assay. **(L)** HK2 thermal stability at 52 °C with different baicalin concentrations (n=3). **(M, N)** Quantification of HK2 levels at 52°C. Data are expressed as mean ± SEM. *p < 0.05, **p < 0.01, ***p < 0.001, ****p < 0.0001.

This study evaluated the *in vitro* anti-proliferative activity of baicalin. Cell viability of HCT116 and MC38 cells, treated with baicalin at concentrations of 0, 10, 20, and 40 μmol/L, was assessed using the CCK-8 assay. Baicalin dose-dependently inhibited the viability of CRC cells (**Figures 1B, C**). Consistently, baicalin markedly reduced colony formation in HCT116 and MC38 cells (**Figure 1D**), further supporting its potent inhibitory effect on CRC cell proliferation. To investigate the underlying mechanism, HK2 protein levels were examined by western blot. Baicalin treatment substantially downregulated HK2 expression (**Figure 1E**). To further assess the impact of baicalin on HK2, the DARTS assay was performed. Following protease treatment, HK2 protein levels exhibited a substantial decrease; however, this decrease was progressively attenuated with increasing concentrations of baicalin (**Figures 1F–H**), indicating increased protease resistance. In addition, CETSA analysis revealed that baicalin markedly enhanced the thermal stability of HK2 (**Figures 1I–K**). Notably, at 52 °C, baicalin increased the abundance of soluble HK2 in a concentration-dependent manner (**Figures 1L–N**), supporting target engagement between baicalin and HK2.

### 3.2 Molecular docking and molecular dynamics simulations of the putative baicalin–HK2 association

**Figure 2:**
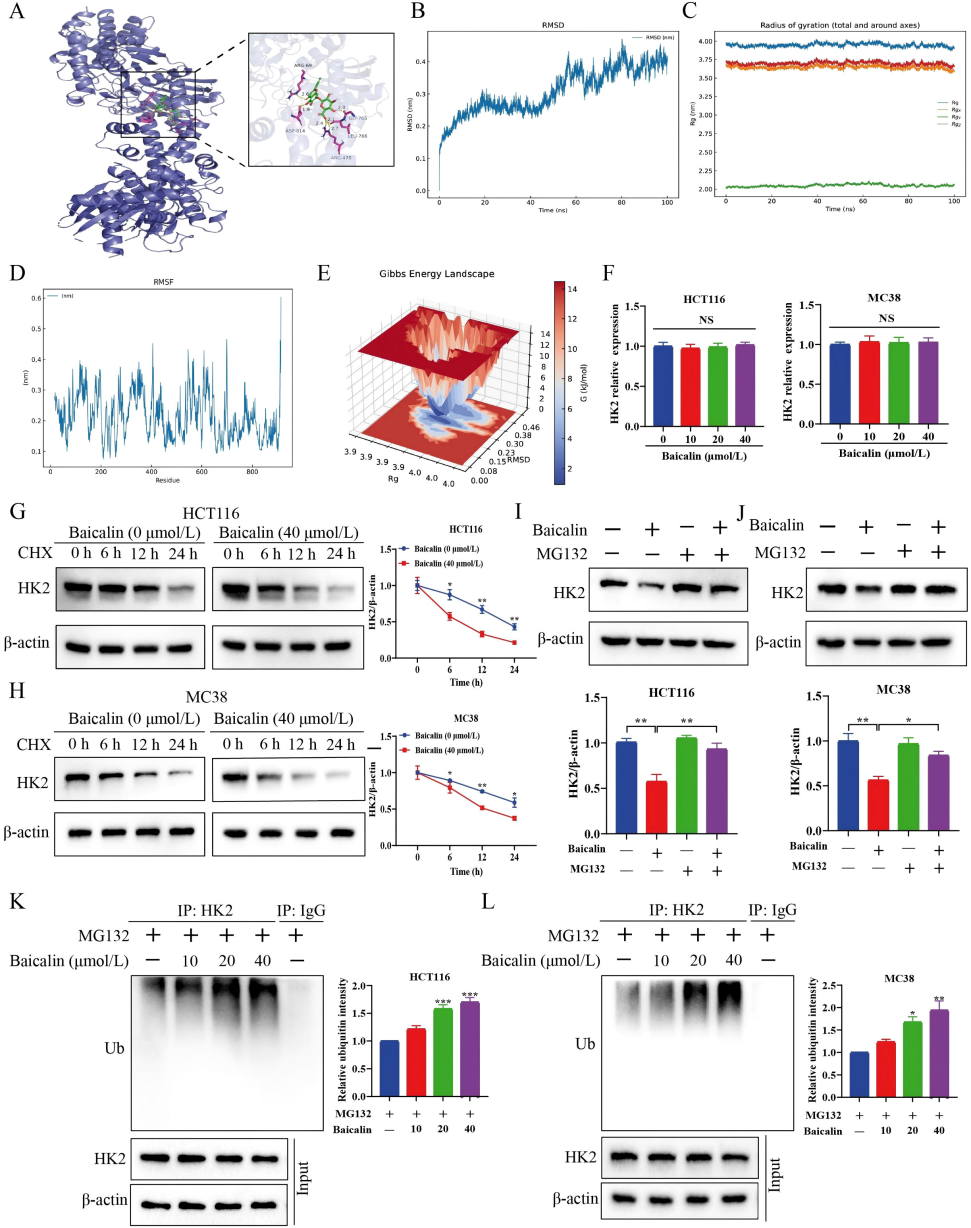
Computational analysis of the putative baicalin–HK2 association and post-translational regulation of HK2 protein levels. **(A)** Molecular docking of baicalin with HK2. **(B)** Root mean square deviation (RMSD). **(C)** Radius of Gyration (Rg). **(D)** Root mean square fluctuation (RMSF). **(E)** The free energy landscape. **(F)** CRC cells were incubated with baicalin at 0, 10, 20, and 40 μmol/L for 24 h. HK2 mRNA expression was measured by RT-qPCR (n=3). **(G, H)** CRC cells were treated with CHX (5 μg/mL) and baicalin (40 μmol/L), and harvested at 0, 6, 12, and 24 h for HK2 protein analysis by western blot (n=3). **(I, J)** Co-treatment with MG132 and baicalin for 24 h attenuated HK2 downregulation in CRC cells (n=3). **(K, L)** Co-IP analysis of HK2 ubiquitination in CRC cells after baicalin treatment (n=3). Data are expressed as mean ± SEM. *p < 0.05, **p < 0.01.

Molecular docking suggested that baicalin may bind to HK2 with a predicted binding energy of -8.957 kcal/mol (**Figure 2A**). To further explore the potential association between baicalin and HK2, molecular dynamics simulations were performed. After an initial equilibration phase within the first 20–40 ns, the RMSD increased beyond ~40 ns and showed pronounced fluctuations in the later stage (approximately in the range of ~0.3–0.45 nm), without a clear convergence trend, suggesting substantial conformational flexibility (**Figure 2B**). Rg and its axial components (Rg/x, Rg/y, Rg/z) remained relatively stable throughout the simulation. Rg fluctuated slightly around ~3.75–4.0 nm, Rg/x and Rg/y were around ~3.5–3.75 nm, and Rg/z was near ~2.0–2.25 nm, indicating that overall structural compactness was well maintained without evidence of global unfolding or collapse (**Figure 2C**). Consistently, the RMSF analysis across the full residue range showed distributed flexibility, with fluctuation amplitudes mainly within ~0.1–0.5 nm, suggesting that while the global fold was preserved, multiple regions retained intrinsic dynamic behavior (**Figure 2D**). Furthermore, the Gibbs free energy landscape revealed a broad low-energy basin rather than a single sharp minimum, with dominant conformational states centered at Rg ≈ 3.9–4.0 nm and RMSD ≈ 0.15–0.46 nm, indicating conformational heterogeneity within a thermodynamically stable ensemble (**Figure 2E**). Collectively, these results indicated that the protein maintained overall structural integrity while displaying pronounced local flexibility, supporting a dynamic conformational ensemble consistent with a binding mode of baicalin to HK2 that likely accommodates conformational variability.

### 3.3 Baicalin promotes the proteasome-mediated degradation of HK2

To clarify how baicalin downregulates HK2 protein levels, we measured HK2 mRNA levels in HCT116 and MC38 cells after baicalin treatment, and found no significant changes (**Figure 2F**). This further suggests baicalin reduces HK2 protein levels post-transcriptionally, independent of mRNA levels. We therefore hypothesize that baicalin affects HK2 protein levels through a post-translational mechanism. Given the central role of the ubiquitin-proteasome pathway in protein degradation, we treated the cells with cycloheximide (CHX) with or without baicalin, and collected samples at 0, 6, 12, and 24 h. CHX alone significantly decreased HK2 protein levels, while co-treatment with baicalin accelerated HK2 degradation (**Figures 2G, H**). This result suggests that baicalin accelerates HK2 degradation. To further investigate the mechanism, cells were treated with the proteasome inhibitor MG132. MG132 largely reversed the baicalin-induced decrease in HK2 protein levels, indicating that baicalin promotes proteasome-dependent degradation of HK2 in CRC cells (**Figures 2I, J**). Furthermore, co-immunoprecipitation (Co-IP) followed by anti-ubiquitin immunoblotting revealed increased ubiquitin signal in HK2 immunoprecipitates, consistent with ubiquitination-associated modification (**Figures 2K, L**). Collectively, these findings support a model of baicalin-induced HK2 degradation through a ubiquitination-associated proteasomal pathway.

### 3.4 High HK2 expression predicts poor prognosis of CRC

**Figure 3:**
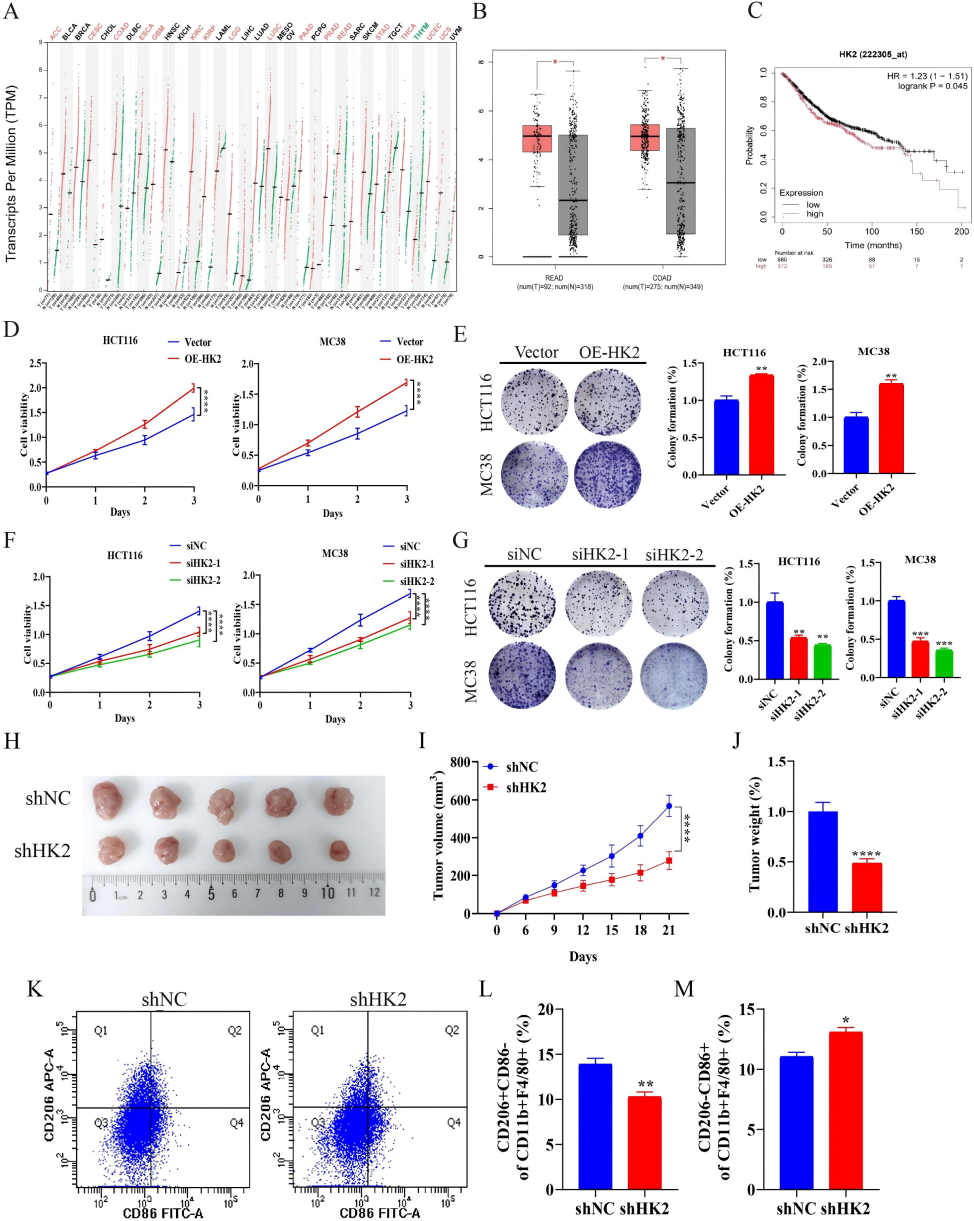
Higher HK2 expression correlates with poor prognosis in CRC. **(A)** Pan-cancer analysis of HK2 mRNA expression using the GEPIA database. **(B)** Box plots comparing HK2 mRNA levels in CRC versus normal tissues. **(C)** Kaplan–Meier survival analysis of overall survival in CRC patients stratified by HK2 expression. **(D, E)** Evaluate the effect of HK2 overexpression on proliferation of HCT116 and MC38 cells using CCK-8 and colony formation assays (n=3). **(F, G)** Evaluate the effect of HK2 knockdown on proliferation of HCT116 and MC38 cells using CCK-8 and colony formation assays (n=3). **(H)** Representative images of tumors at the experimental endpoint (n=5). **(I, J)** Tumor growth curves and tumor weight (n=5). **(K–M)** Flow cytometry quantification of macrophage populations in the tumor microenvironment (n=3). Data are expressed as mean ± SEM. *p < 0.05, **p < 0.01, ***p < 0.001, ****p < 0.0001.

Previous studies have demonstrated that HK2 is critical for tumor initiation and progression, and that HK2 inhibition has emerged as a promising therapeutic approach across multiple cancer types (32, 33). We first examined HK2 mRNA expression using the GEPIA database. HK2 levels were notably elevated in CRC tissues relative to normal tissues (**Figures 3A, B**). Kaplan–Meier survival analysis indicated that elevated HK2 expression was associated with poorer prognosis in CRC, demonstrating significantly reduced overall survival in the high-expression group (**Figure 3C**).

To further elucidate the function of HK2 in CRC, we generated stable HK2-overexpressing HCT116 and MC38 cells. HK2 overexpression markedly increased proliferation and colony formation of CRC cells (**Figures 3D, E**). Conversely, siRNA-mediated HK2 knockdown markedly suppressed proliferation and colony formation of CRC cells (**Figures 3F, G**). We also established an MC38 cell line with stable shRNA-mediated HK2 silencing (shHK2) and implanted shHK2 and shNC cells subcutaneously into C57BL/6 mice. Under identical experimental conditions, tumors in the shHK2 group were markedly smaller than those in the shNC group (**Figures 3H–J**). In parallel, flow cytometry of tumor tissues showed a significant reduction in CD206+CD86- macrophages and a significant increase in CD206-CD86+ macrophages in the shHK2 group (**Figures 3K–M**). Collectively, these results show that HK2 promotes CRC progression and reshapes tumor-associated macrophage polarization.

### 3.5 HK2 inhibition suppresses glycolysis and induces mitochondrial damage

**Figure 4:**
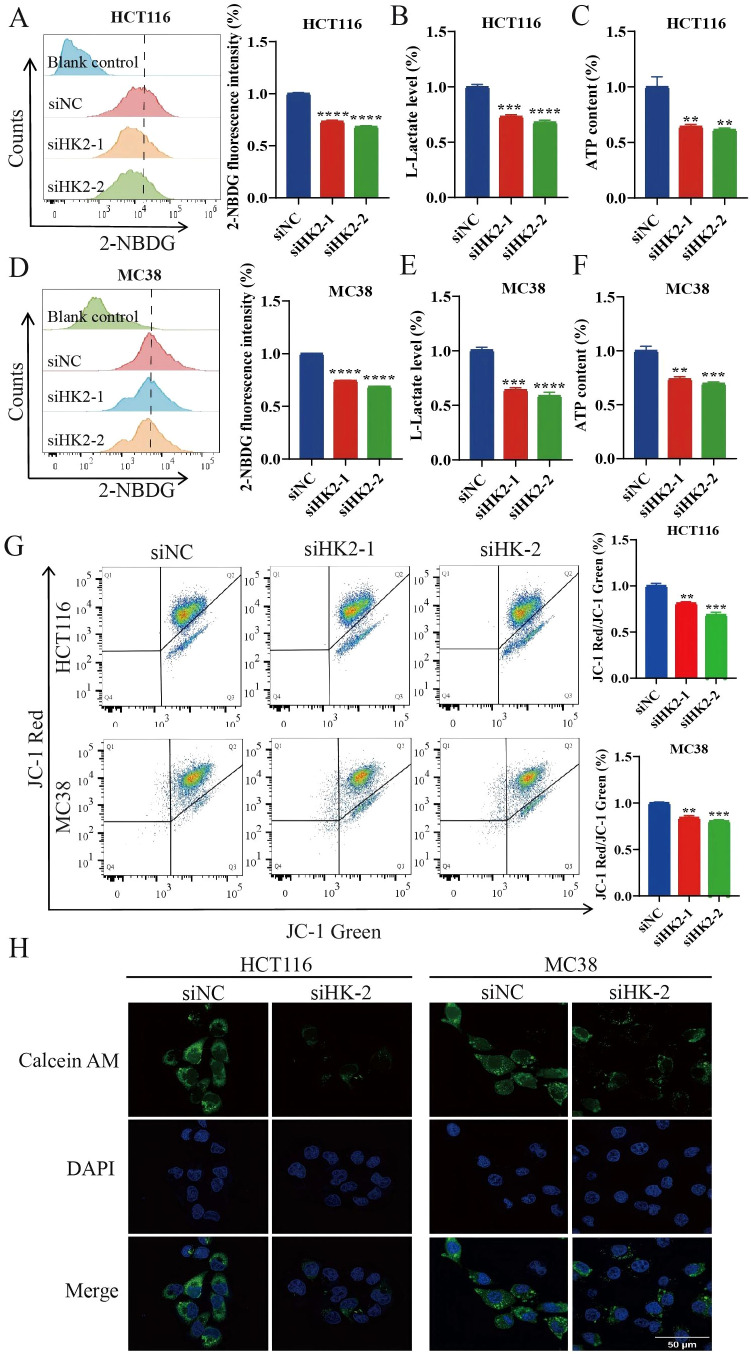
HK2 inhibition suppresses glycolysis and induces mitochondrial damage. **(A, D)** Glucose uptake (n=3). **(B, E)** L-lactate levels (n=3). **(C, F)** ATP content (n=3). **(G)** Mitochondrial membrane potential (n=3). **(H)** mPTP (n=3). Scale bar, 50 μm. Data are expressed as mean ± SEM. **p < 0.01. ***p < 0.001, ****p < 0.0001.

Beyond its canonical role in glucose metabolism, HK2 also attaches to the outer mitochondrial membrane and binds to VDAC. This interaction helps maintain mitochondrial membrane potential, thereby supporting mitochondrial function (34–36). To further define the contribution of HK2 knockdown in CRC cells, we knocked down HK2 in HCT116 and MC38 cells and examined glucose uptake, L-lactate production, and ATP content. HK2 knockdown markedly impaired glycolytic metabolism, as evidenced by reduced glucose uptake, decreased L-lactate production, and decreased ATP content, suggesting that HK2 is a key regulator of glycolysis in CRC cells (**Figures 4A–F**). We next assessed mitochondrial function and found that HK2 knockdown significantly decreased mitochondrial membrane potential and facilitated mPTP opening, indicating that HK2 inhibition induces mitochondrial dysfunction (**Figures 4G, H**). These findings indicate that HK2 knockdown can suppress CRC cell proliferation by concurrently regulating glycolysis and inducing mitochondrial dysfunction.

### 3.6 HK2 inhibition activates the cGAS/STING pathway and promotes M1 polarization of tumor-associated macrophages

**Figure 5:**
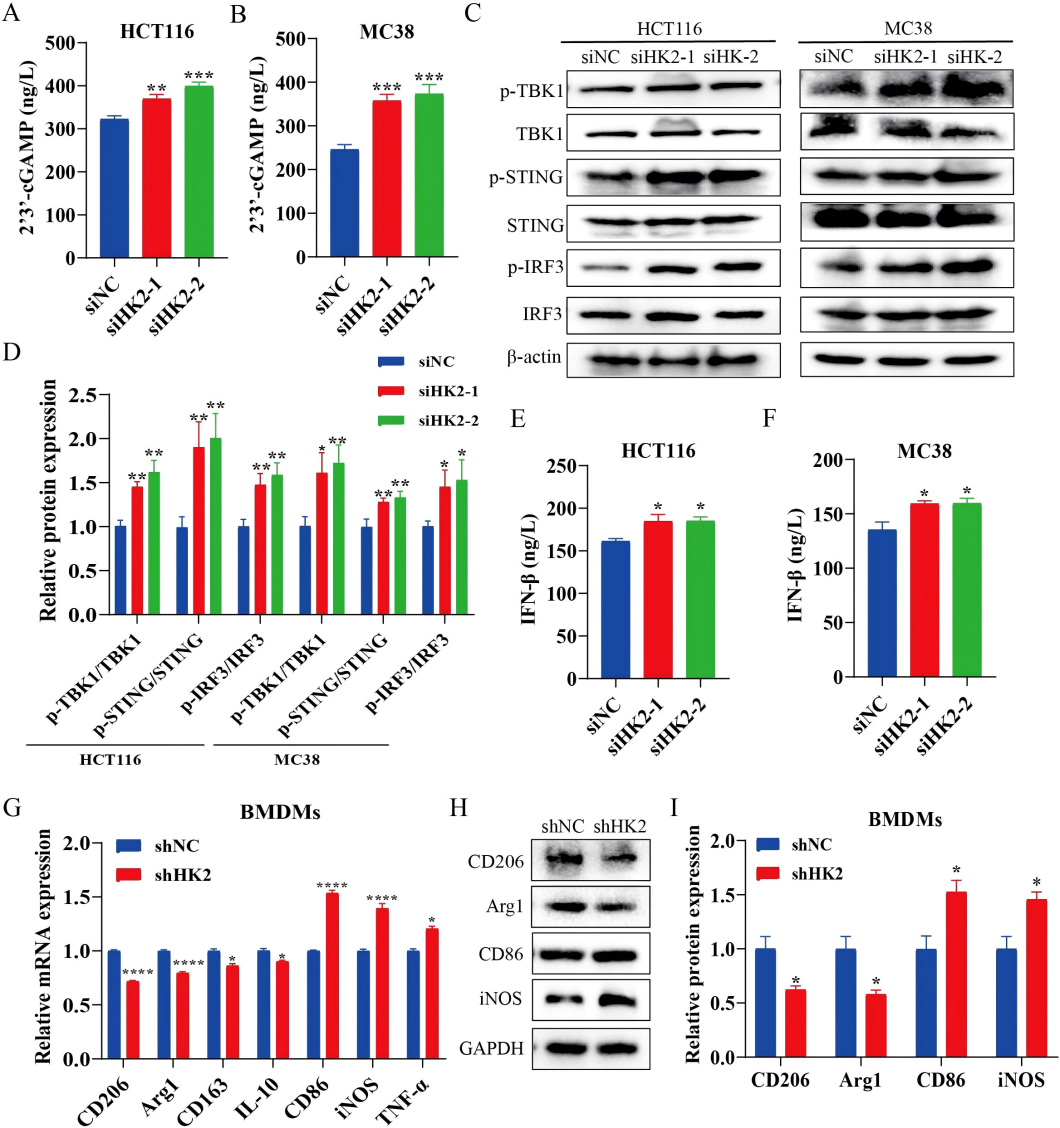
HK2 inhibition activates the cGAS/STING pathway and promotes M1 polarization of tumor-associated macrophages. **(A, B)** Intracellular 2’3’-cGAMP levels (n=3). **(C)** Western blot analysis of cGAS/STING pathway proteins (n=3). **(D)** Quantification of cGAS/STING pathway proteins (n=3). **(E, F)** IFN-β levels in culture supernatants (n=3). **(G)** RT–qPCR analysis of polarization-related marker mRNA in BMDMs (M1: CD86, iNOS, and TNF-α; M2:CD206, Arg1, CD163, and IL-10) (n=3). **(H)** Western blot validation of polarization-associated protein changes in BMDMs (M1: CD86, iNOS; M2: CD206, Arg1) (n=3). **(I)** Quantification of polarization-associated protein levels in BMDMs (n=3). Data are expressed as mean ± SEM. *p < 0.05, **p < 0.01. ***p < 0.001, ****p < 0.0001.

Mitochondrial damage can release mtDNA into the cytosol, increasing cytosolic dsDNA. Accumulated cytosolic dsDNA activates the cGAS/STING pathway and drives IFN-β production (37, 38). Because the impact of HK2 in modulating cGAS/STING signaling remains unclear, we investigated whether HK2 inhibition affects this pathway. We first observed that HK2 knockdown increased intracellular 2’3’-cGAMP levels in CRC cells (**Figures 5A, B**). Consistent with cGAS/STING activation, HK2 inhibition enhanced phosphorylation of key signaling components, including TBK1, STING, and IRF3 (**Figures 5C, D**). Accordingly, IFN-β concentrations in the culture supernatant were markedly elevated (**Figures 5E, F**).

Macrophages are central immune cells within the tumor microenvironment, and accumulating evidence indicates that the cGAS/STING signaling pathway can regulate the polarization of tumor-associated macrophages, thereby influencing anti-tumor immune responses (39, 40). We next established a tumor-associated macrophage model by co-culturing MC38 cells with BMDMs. RT–qPCR analysis showed that co-culture with HK2-knockdown MC38 cells significantly modulated M1/M2-associated markers in BMDMs (**Figure 5G**). Western blot analysis further corroborated these findings, showing decreased CD206 and Arg1 levels with increased CD86 and iNOS expression (**Figures 5H, I**), suggesting that HK2 inhibition promotes a shift in tumor-associated macrophages toward an M1-like polarization state.

### 3.7 Baicalin suppresses HK2-mediated glycolysis and promotes cGAS/STING pathway

**Figure 6:**
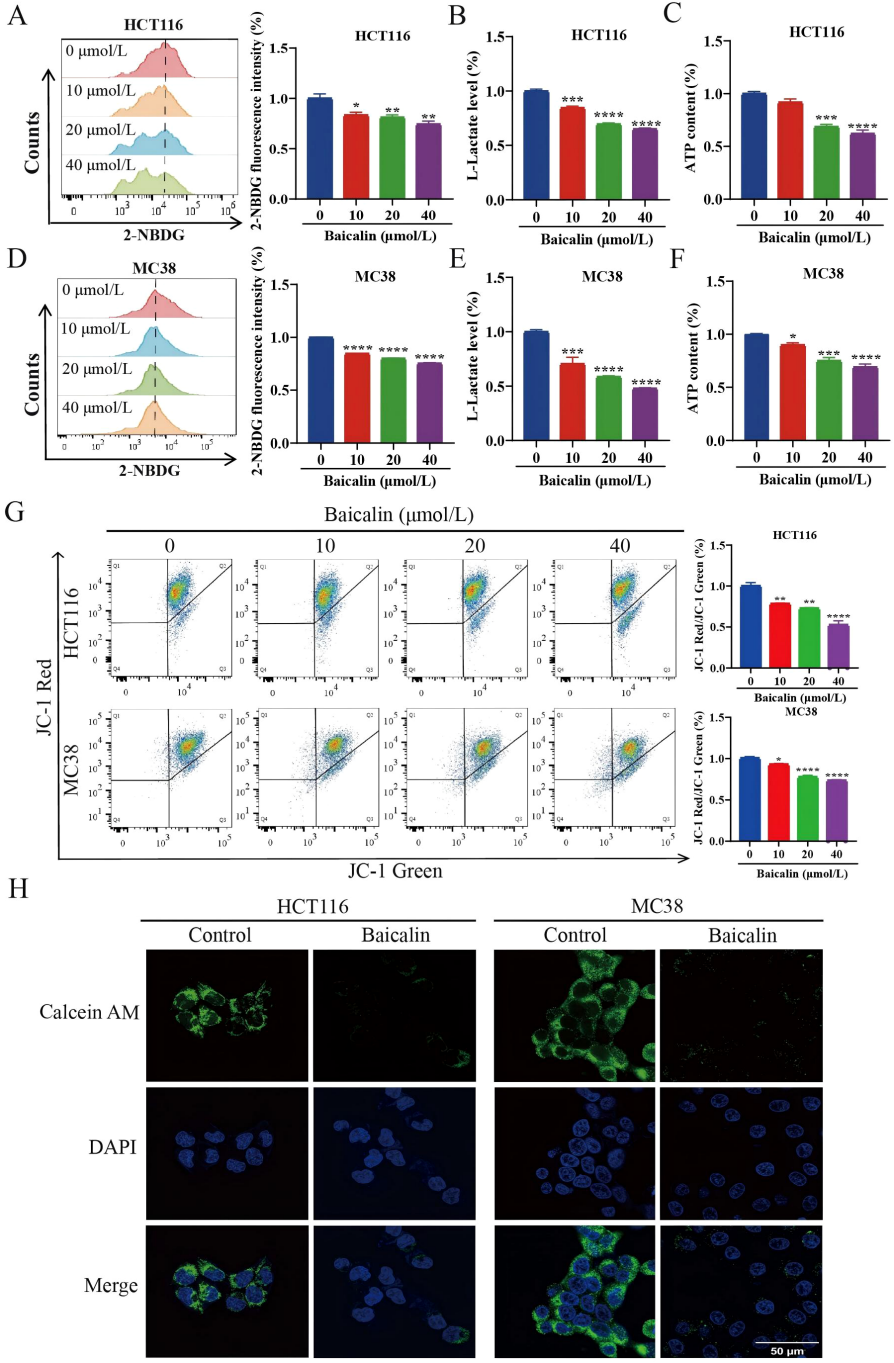
Effects of baicalin on glycolysis and mitochondrial dysfunction in CRC cells. **(A, D)** Glucose uptake (n=3). **(B, E)** L-lactate levels (n=3). **(C, F)** ATP content (n=3). **(G)** Mitochondrial membrane potential (n=3). **(H)** mPTP (n=3). Scale bar, 50 μm. Data are expressed as mean ± SEM. *p < 0.05, **p < 0.01, ***p < 0.001, ****p < 0.0001.

**Figure 7:**
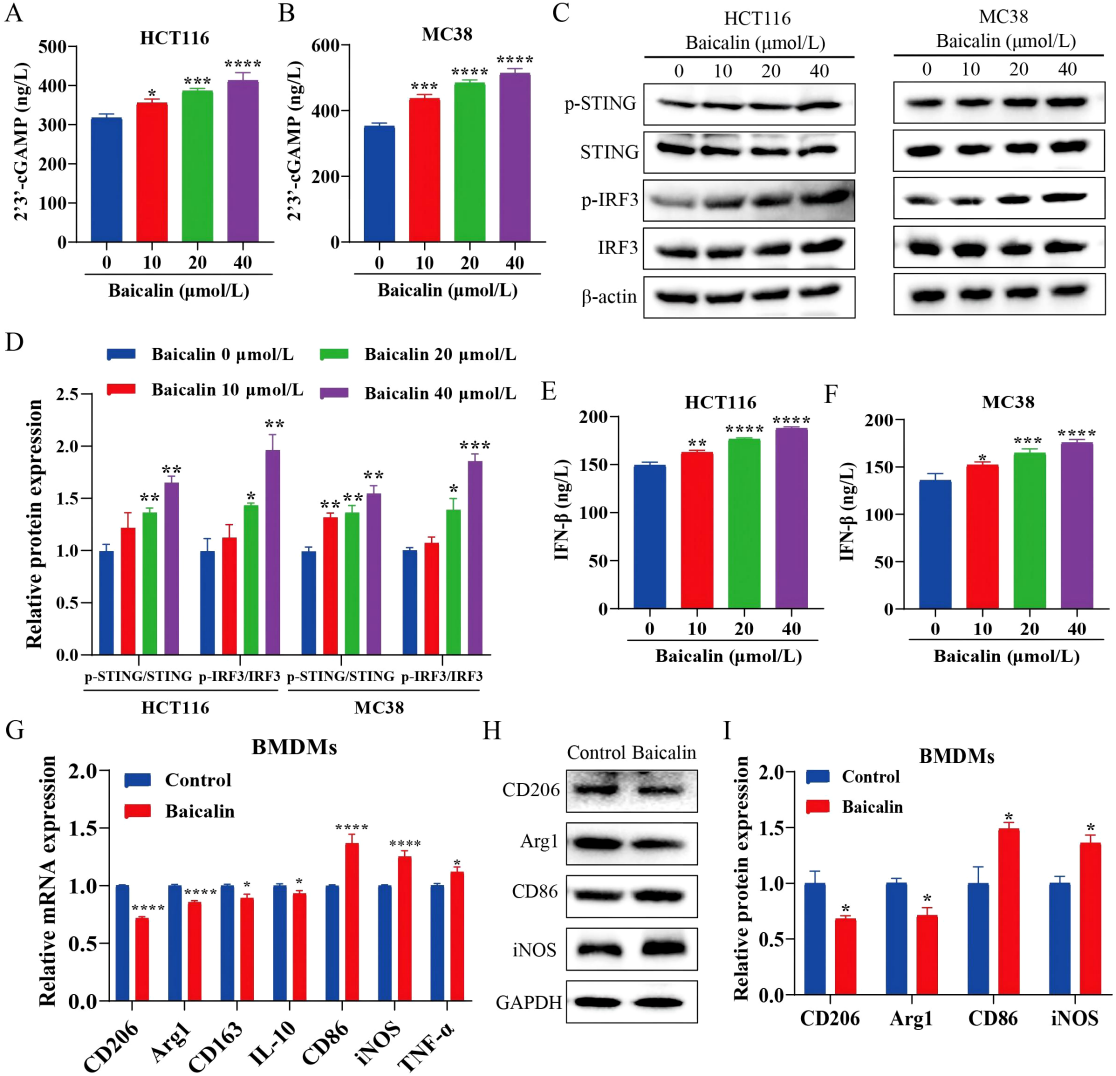
Baicalin activates the cGAS/STING signaling pathway and reverses tumor-associated macrophage polarization. **(A, B)** Intracellular 2’3’-cGAMP levels (n=3). **(C)** Western blot analysis of cGAS/STING pathway proteins (p-STING/STING and p-IRF3/IRF3) (n=3). **(D)** Quantification of cGAS/STING pathway protein levels (n=3). **(E, F)** IFN-β levels in culture supernatants (n=3). **(G)** RT–qPCR analysis of polarization-related marker mRNA in BMDMs (M1: CD86, iNOS, and TNF-α; M2: CD206, Arg1, CD163, and IL-10) (n=3). **(H)** Western blot validation of polarization-associated protein changes in BMDMs (M1: CD86, iNOS; M2: CD206, Arg1) (n=3). **(I)** Quantification of polarization-associated protein levels in BMDMs (n=3). Data are expressed as mean ± SEM. *p < 0.05, **p < 0.01. ***p < 0.001, ****p < 0.0001.

Building on our previous finding that baicalin inhibits HK2, we further evaluated its effects on glycolysis and the cGAS/STING pathway in CRC cells. Baicalin treatment significantly decreased glucose uptake, reduced L-lactate release, and lowered ATP content in CRC cells (**Figures 6A–F**), indicating a marked suppression of glycolytic activity. Assessment of mitochondrial function showed that baicalin diminished the mitochondrial membrane potential and enhanced mPTP opening (**Figures 6G, H**). Meanwhile, western blot analysis revealed increased phosphorylation of STING and IRF3, accompanied by elevated 2’3’-cGAMP and IFN-β levels, supporting activation of the cGAS/STING signaling pathway (**Figures 7A–F**). Meanwhile, co-culturing BMDMs with baicalin-treated MC38 cells notably modulated M1/M2-associated markers in BMDMs (**Figures 7G–I**). Further studies showed that HK2 overexpression can reverse the effects of baicalin on CRC cell proliferation and tumor-associated macrophage polarization, demonstrating that the effects of baicalin on CRC are dependent on HK2 (**Supplementary Figure 2**). Taken together, our results indicate that baicalin could activate cGAS/STING by inhibiting HK2-driven glycolysis and inducing mitochondrial dysfunction.

### 3.8 Baicalin suppresses syngeneic tumor growth by inhibiting HK2 and promotes M1 polarization of tumor-associated macrophages

**Figure 8:**
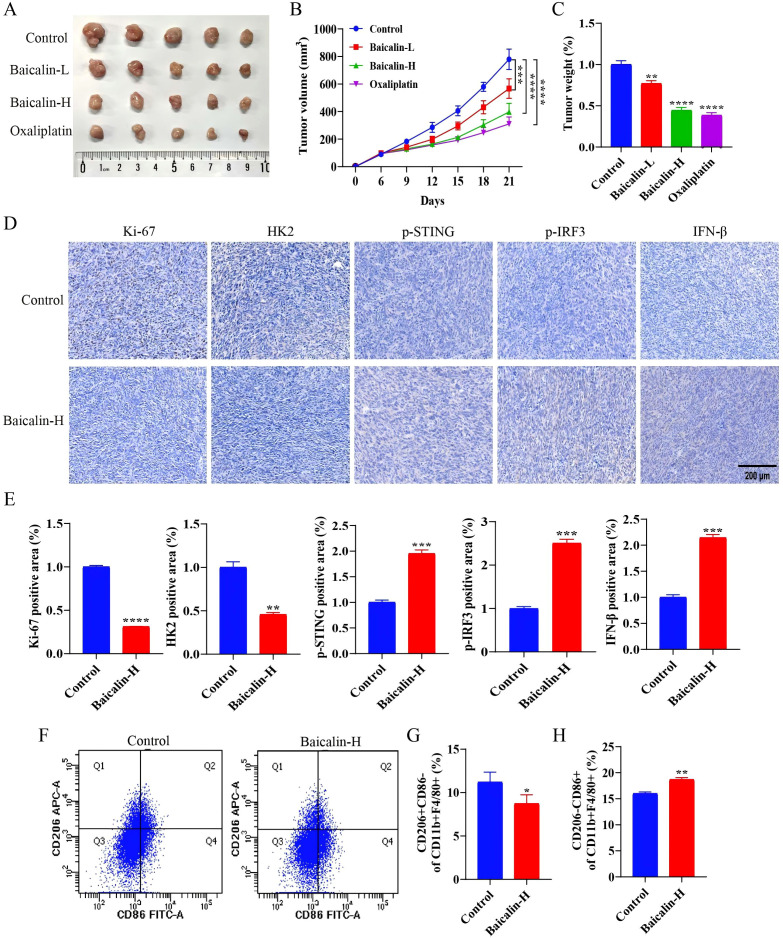
Baicalin reduced the tumor growth *in vivo*. **(A)** Representative images of tumors at the experimental endpoint (n=5). **(B, C)** Tumor growth curves and endpoint tumor weight. **(D)** Immunohistochemistry was used to assess the expression levels of Ki-67, HK2, p-STING, p-IRF3, and IFN-β in tumor tissues (n=3). Original magnification: × 40. **(E)** Quantitative analysis of Ki-67, HK2, p-STING, p-IRF3, and IFN-β expression in tumor tissues was performed using ImageJ software (n=3). **(F–H)** Flow cytometry quantification of macrophage populations in the tumor microenvironment (n=3). Data are expressed as mean ± SEM. *p < 0.05, **p < 0.01. ***p < 0.001, ****p < 0.0001.

We established a subcutaneous syngeneic tumor model to assess the *in vivo* antitumor efficacy of baicalin. Baicalin markedly suppressed tumor growth (**Figures 8A, B**) and decreased tumor weight (**Figure 8C**). Immunohistochemistry revealed that baicalin treatment reduced Ki-67 and HK2 expression in tumor tissues, while increasing the expression of p-STING, p-IRF3, and IFN-β (**Figures 8D, E**). Flow cytometry analysis further showed a decreased proportion of CD206+CD86- macrophages and an increased proportion of CD206-CD86+ macrophages in tumor tissues (**Figures 8F–H**). Collectively, these *in vivo* findings indicate that baicalin suppresses tumor cell proliferation by inhibiting HK2 and activates cGAS/STING signaling to promote a shift in tumor-associated macrophages toward an M1-like polarization state.

## 4 Discussion

Glycolysis inhibitors, as a new therapeutic strategy, have shown potential in the treatment of cancer. However, most glycolysis inhibitors are still in clinical trials or preclinical stages (36, 41, 42). Glycolysis is primarily regulated by key rate-limiting enzymes, including HK2, PKM2, and LDHA (43, 44). In this study, we found that the naturally derived compound baicalin may serve as a potential HK2 inhibitor, thereby suppressing the progression of colorectal cancer. CCK-8 assay and colony formation assays demonstrated that baicalin dose-dependently reduced cell viability and colony formation, indicating a potent anti-proliferative effect *in vitro*. Mechanistically, baicalin markedly decreased HK2 protein levels, while DARTS and CETSA assays showed increased proteolytic stability and thermal stability of HK2 upon baicalin treatment, thereby supporting target engagement between baicalin and HK2 in cells. In line with these findings, molecular docking and molecular dynamics simulations indicated a potential association between baicalin and HK2, warranting further investigation. Baicalin did not affect HK2 mRNA expression, but accelerated HK2 protein degradation. Notably, the proteasome inhibitor MG132 largely reversed baicalin-induced HK2 downregulation, and Co-IP assays showed baicalin treatment increased HK2 ubiquitination, suggesting that ubiquitination-associated proteasomal degradation is involved in the reduction of HK2 protein levels. Previous studies have shown that, beyond its traditional function in glycolysis, HK2 can localize to the mitochondrial outer membrane and interact with VDAC, thereby contributing to the maintenance of mitochondrial homeostasis (45, 46). Based on GEPIA database analyses and Kaplan–Meier survival analysis, HK2 expression is markedly upregulated in CRC tissues, and high HK2 expression is associated with shorter overall survival. *In vitro* experiments further confirmed that HK2 overexpression significantly enhanced CRC cell proliferation, whereas HK2 knockdown significantly inhibited these phenotypes. Tumors in the shHK2 group were significantly smaller compared to the shNC group. In parallel, flow cytometry of tumor tissues showed a significant reduction in M2-like macrophages in the shHK2 group. Collectively, these findings indicate that HK2 promotes CRC progression and reshapes tumor-associated macrophage polarization. Further mechanistic analyses revealed that HK2 knockdown markedly decreased glucose uptake, L-lactate production, and ATP content, confirming effective inhibition of glycolysis. Concomitantly, HK2 knockdown induced a decrease in mitochondrial membrane potential and promoted mPTP opening. Given that mitochondrial injury can promote mtDNA release and the accumulation of cytosolic dsDNA, thereby activating the cGAS/STING pathway, we evaluated this signaling axis and found that HK2 suppression increased 2’3’-cGAMP levels, enhanced phosphorylation of STING, TBK1, and IRF3, and increased IFN-β secretion. Moreover, in a TAM model established by co-culturing MC38 cells with BMDMs, HK2 inhibition decreased M2-associated markers and increased M1-associated markers. Together, these findings indicate that HK2 couples glycolytic metabolism and mitochondrial homeostasis to promote activation of the cGAS/STING pathway, thereby reprogramming macrophage polarization.

Baicalin suppressed HK2-mediated glycolysis and induced mitochondrial damage, thereby activating the cGAS/STING pathway and promoting TAM repolarization from an M2-like toward an M1-like phenotype. Consistently, in an MC38 syngeneic tumor model, baicalin exerted *in vivo* antitumor effects, significantly inhibiting tumor growth and enhancing M1-like polarization of tumor-associated macrophages. Immunohistochemistry revealed that baicalin treatment reduced HK2 expression in tumor tissues, while increasing p-STING, p-IRF3, and IFN-β levels, indicating that baicalin inhibits HK2 and activates the cGAS/STING signaling axis *in vivo*.

## 5 Conclusion

This study is the first to show that baicalin inhibits the proliferation of CRC cells and promotes a shift in tumor-associated macrophages toward an M1-like polarization state. Mechanistically, baicalin engages HK2 and promotes its proteasome-dependent degradation, which is consistent with ubiquitin-mediated modification. This inhibits glycolysis, induces mitochondrial damage, and suppresses CRC cell proliferation. Further studies reveal that baicalin-induced mitochondrial damage activates the cGAS/STING signaling pathway, thereby promoting a shift in tumor-associated macrophages toward an M1-like polarization state. This study uncovers a novel strategy for inhibiting HK2 to simultaneously regulate both tumor cell metabolism and the immune microenvironment, suggesting baicalin as a potential natural therapeutic candidate and providing a basis for targeted CRC treatment.

## Data Availability

The datasets presented in this study can be found in online repositories. The names of the repository/repositories and accession number(s) can be found in the article/**Supplementary Material**.
