# Supplementary material for: Baicalin suppresses colorectal cancer proliferation and induces M1 polarization of tumor-associated macrophages by promoting proteasomal degradation of HK2
Source: Front Immunol. 2026 May 4;17:1812964. doi: 10.3389/fimmu.2026.1812964 (PMC13180920; doi:10.3389/fimmu.2026.1812964)
Supplement: Supplementary file 1 [file DataSheet1.docx]

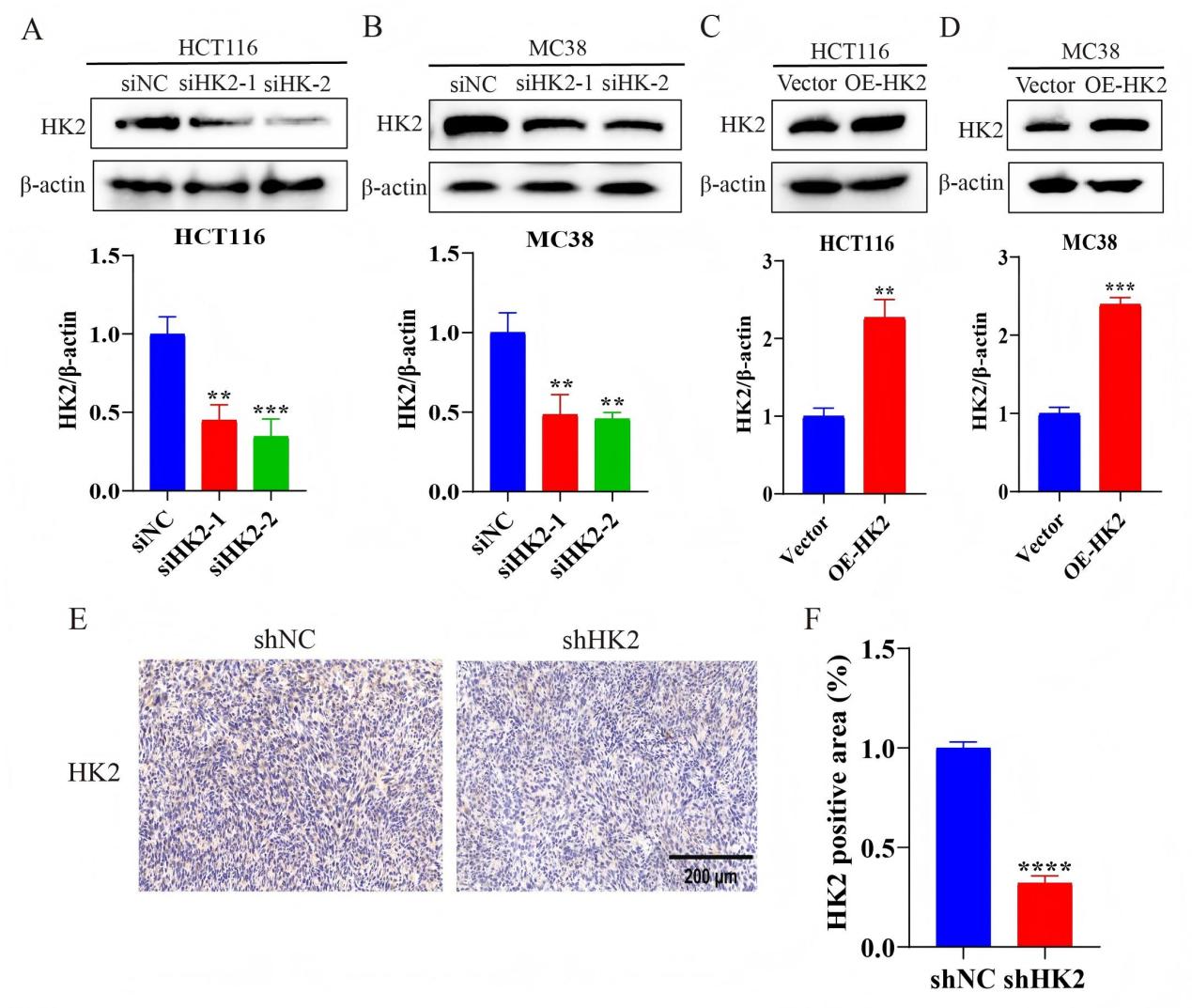
Supplementary figure. 1 Western blot validation of HK2 knockdown and overexpression efficiency in CRC cells. (A–B) Western blot analysis confirming HK2 knockdown in CRC cells; (C–D) Western blot analysis confirming HK2 overexpression in CRC cells; (E) Immunohistochemical analysis of HK2 in graft tumors; (F) Quantitative analysis of HK2 expression in tumor tissues. Data are presented as mean ± SEM. ***p* < 0.01, ****p* < 0.001, *****p* < 0.0001.


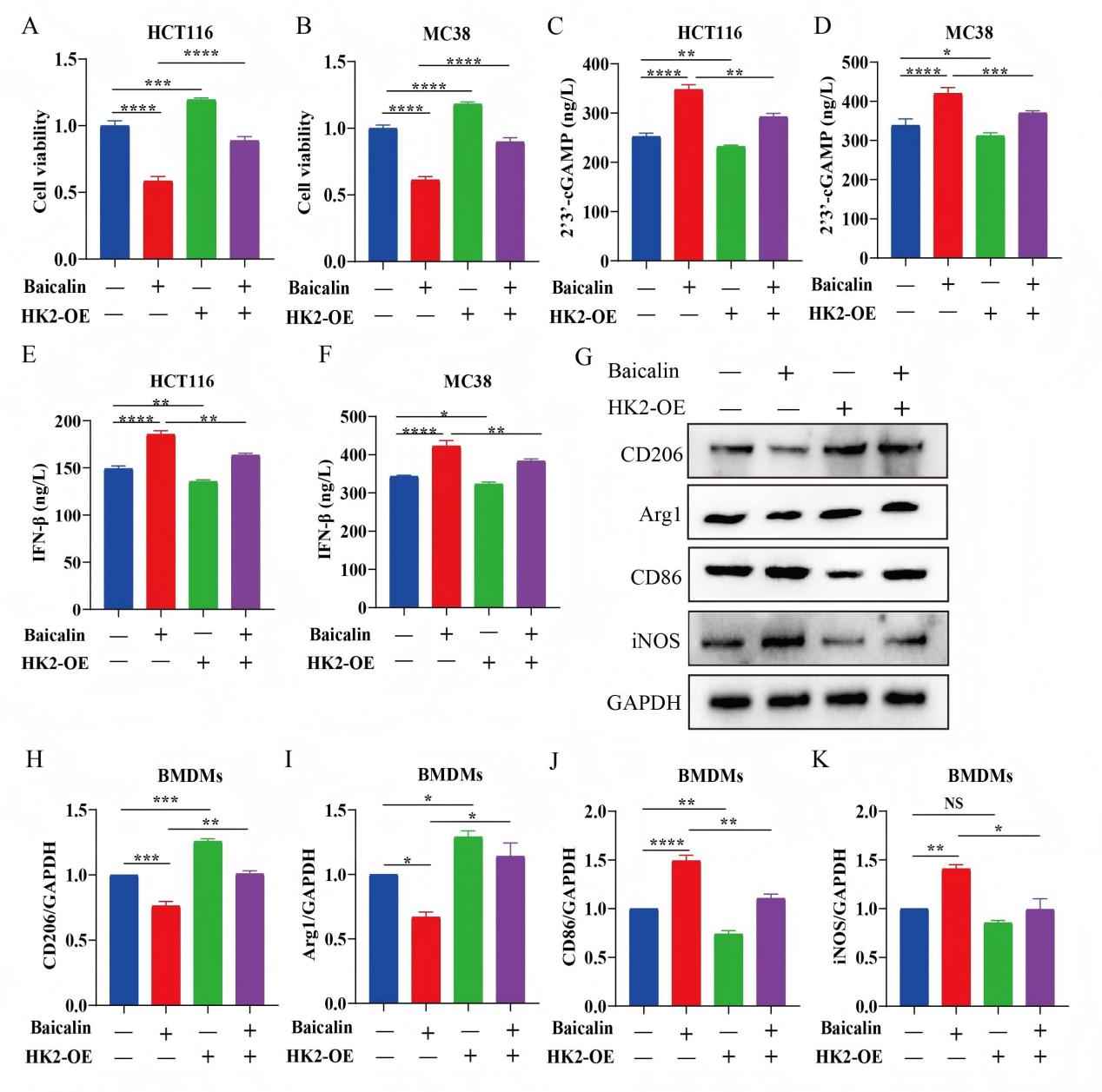


Supplementary figure. 2 Overexpression of HK2 reverses the effects of baicalin on CRC. (A-B) Overexpression of HK2 significantly reverses the inhibitory effect of baicalin on HCT116 and MC38 cell proliferation. (C-D) Overexpression of HK2 reverses the activation of 2’3’-cGAMP production induced by baicalin in HCT116 and MC38 cells. (E-F) Overexpression of HK2 reverses the promotion of IFN-β production induced by baicalin in HCT116 and MC38 cells. (G) Overexpression of HK2 reverses the regulatory effects of baicalin on tumor-associated macrophages. (H-K) Quantification of protein levels in BMDM cells. Data are presented as mean ± SEM, **p* < 0.05, ***p* < 0.01, ****p* < 0.001, *****p* < 0.0001.


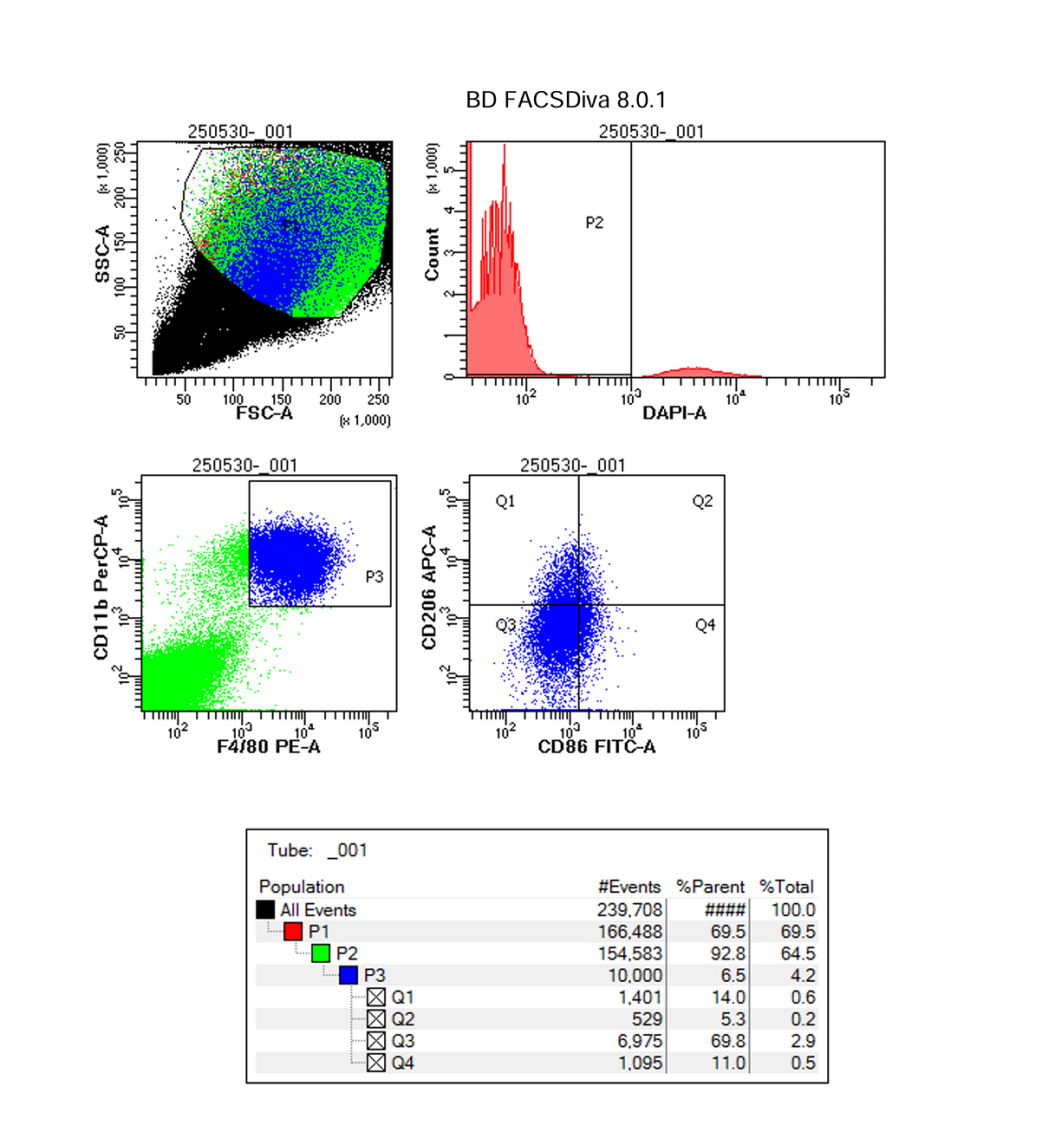


Supplementary figure 3. Gating strategy for macrophages in tumor tissue.
